# An mHealth Stress Management Intervention for Latino Millennial Caregivers: Pilot Feasibility Randomized Controlled Trial

**DOI:** 10.2196/85396

**Published:** 2026-10-27

**Authors:** Sandi Alshahwan, Megan Thomas Hebdon, Cheongin Im, Michael Thomas, Neil Peterson, Janice Hernandez, Heather Cuevas, Sharon Horner

**Affiliations:** 1 College of Nursing, University of Utah, 10 S 2000 E, Salt Lake City, UT, 84112, United States, 1 8019135863; 2 Department of Nursing, College of Medicine, Catholic Kwandong University, Gangneung-si, Gangwon-do, Republic of Korea; 3 College of Nursing, Brigham Young University, Provo, UT, United States; 4 School of Nursing, The University of Texas at Austin, Austin, TX, United States

**Keywords:** Latino caregivers, millennials, mobile health intervention, mHealth intervention, stress management, mindfulness, journaling, cultural tailoring

## Abstract

**Background:**

Latino millennial caregivers comprise over one-quarter of millennial family caregivers in the United States. Latino millennial family caregivers face unique challenges due to cultural, structural, and economic burdens that may increase stress and stress-related health effects. These caregivers also report time limitations due to their multiple roles. Technology may be a way to deliver an accessible and flexible intervention to support stress management and emotional regulation in this population.

**Objective:**

This study aimed to evaluate the feasibility, acceptability, and preliminary outcomes of a culturally tailored mobile health (mHealth) intervention to support stress management and emotional regulation for Latino millennial caregivers.

**Methods:**

Participants (N=30) were recruited nationally in the United States using a recruitment agency and were randomized to the intervention or waitlist control groups. The 4-week fully automated intervention included daily mindfulness exercises and journaling prompts delivered via a custom mHealth platform. The waitlist control group received a written handout with mindfulness, journaling, and support information. Feasibility data, including recruitment and retention rates and intervention adherence, and postintervention acceptability surveys were collected. Study outcome measures were collected electronically at baseline and 2, 4, and 6 weeks. Primary outcomes included caregiver burden and perceived stress, and secondary outcomes included well-being, informational support, and emotional support. Feasibility and acceptability data were analyzed descriptively alongside study outcome measure data from participants who completed week 6 data collection (n=21). No efficacy testing was conducted.

**Results:**

Enrollment was completed in 12 weeks (N=30), with a 70% (21/30) retention. Participants showed differential adherence, with 60% (6/10) completing a daily journal activity and 30% (3/10) completing a daily mindfulness activity. Adherence patterns were reflected in the acceptability findings, with journaling being preferred over mindfulness. Preliminary outcomes showed varying patterns between the intervention and control groups, with the intervention group showing decreased median burden levels from before (median 20, IQR 15-27.25) to after the intervention (median 15, IQR 10.50-28.75) but increased median stress levels from 19.50 (IQR 12-26.75) to 21 (IQR 8.75-22.75), while the control group burden and stress levels remained the same before and after the intervention. Positive affect and well-being and informational support increased for both groups. Median emotional support levels increased from 15 (IQR 12-20) before the intervention to 16 (IQR 13-19) after the intervention for the control group and decreased from 14.5 (IQR 11.5-17.75) to 14 (IQR 12-17.25) for the intervention group.

**Conclusions:**

Findings demonstrate areas for further intervention iteration to address both adherence and acceptability issues with the mindfulness component of the intervention. Intervention implementation also needs to be addressed, including participant retention rates. Our team will use lessons learned alongside feedback from Latino millennial caregivers to address these issues.

## Introduction

Family caregivers of family members, neighbors, and friends with chronic illnesses and disabilities are the economic and care backbone of our health care system [1,2]. Millennial family caregivers are those born between 1981 and 1996 and comprise 25% of caregivers in the United States [3]. Millennial family caregivers have unique needs specific to their generational experiences and development stage [4-7]. Broadly, the millennial generation has experienced significant economic setbacks, including two global recessions and the COVID-19 pandemic [7]. These economic challenges have contributed to delayed important developmental milestones for millennial caregivers, such as educational progression, career development, home ownership, and retirement savings [8]. Millennials are also more diverse than previous generations across gender identity, sexual orientation, race, and ethnicity, and in the United States, there continue to be inequities in social and health outcomes among minoritized groups [6,9]. In addition to family caregiving, which is stressful, millennial family caregivers navigate complex economic, developmental, and social issues that impact their caregiving experiences [10].

Approximately 27% of millennial caregivers identify as Latino [4]. For the Latino millennial caregiver population, there are significant socioeconomic and health care barriers in the United States due to immigration policies and historic and current practices that reinforce bias and segregation [11,12]. There continue to be issues in the health care system with language access and lack of cultural alignment between social and health care resources and Latino family-centered cultural expectations [11,13-15]. Many within this group experience poverty and limited career prospects, which add to the stress of caregiving [16,17]. Additionally, Latino caregivers contribute a higher percentage of their income toward caregiving expenses compared to their non-Latino peers despite often holding lower-income jobs than their White, non-Hispanic counterparts [3]. These financial burdens, combined with the physical and emotional toll of caregiving, expose them to a higher risk of adverse health outcomes [17,18]. Finally, among Latino millennial caregivers, there are additional layers of stress due to cultural expectations such as *familismo* (loyalty to family) and gendered expectations (*marianismo* and machismo) that may impact how the caregiving role is experienced regardless of gender [11,13,19].

Family caregivers experience acute and chronic stress that can contribute to a wide range of adverse health effects, including mental health issues, compromised quality of life, and elevated mortality [20]. Myruski et al [21] introduced the dynamic fit model as an approach to understanding acute and chronic stress. They propose that there are in-the-moment responses to high acute stress and sustained responses to lower-level chronic stress. They posit that well-being is best achieved when coping strategies are aligned with the type of stress. Mindfulness is a stress management and emotional regulation tool that emphasizes being in the moment, and it can be deployed readily without the need for specific tools or change in location [22]. Mindfulness techniques such as mindful breathing, meditation, and guided reflection exercises can induce emotional regulation, leading to a decrease in stress and depressive symptoms [23]. Through practice, mindfulness can be an orientation to life events that can facilitate stress management and emotional regulation in the moment [22]. To address sustained stress and deliberative coping, narrative interventions (ie, expressive writing, journaling, and gratitude journal) promote the ability to communicate life experiences, whether positive or negative, and have been linked to enhanced stress management and well-being and lower depression in family caregivers and other populations [24-27].

For family caregivers who are juggling multiple responsibilities such as work and family, the accessibility of interventions is of utmost concern. In our prior work with millennial caregivers, both overall millennial caregivers and Latino millennial caregivers noted the demands on their time [8,16,28]. Digital health interventions, especially mobile health (mHealth) interventions, present promising solutions by offering scalable, accessible, and flexible support tailored to caregivers’ needs [29-31]. mHealth interventions have been found to be effective in providing support, enhancing communication, and increasing self-efficacy among caregivers [32]. Given the unique challenges faced by Latino millennial caregivers, it is crucial to develop interventions that are culturally sensitive and directly address their specific needs [16].

The purpose of this study was to evaluate the feasibility, acceptability, and preliminary outcomes of a stress management and emotional regulation mHealth intervention for Latino millennial caregivers. Findings from this study will be used to inform further intervention iteration.

## Methods

### Study Design

A 6-week pilot feasibility and acceptability study (4 weeks of active intervention—baseline to 4 weeks) was conducted to test an mHealth-delivered intervention focused on stress management and emotional regulation for Latino millennial family caregivers using an attention, waitlist control approach. Our goal was to ensure that all participants were eventually granted access to the intervention. The protocol was registered in the Open Science Framework retrospectively to align with publishing standards (digital object identifier 10.17605/OSF.IO/B62NH). Prospective registration was not sought due to the pilot nature of our study. We followed the CONSORT-EHEALTH (Consolidated Standards of Reporting Trials of Electronic and Mobile Health Applications and Online Telehealth) guidelines and have included a checklist (Checklist 1).

During consent, participants were told about the study activities they might engage in, but they were not specifically identified as intervention or control activities. After consent procedures, participants were randomized to the treatment or control group using a computer-based random number generator. Participants were blinded to their group assignment, but the study team was not. Participants were assigned a study ID number and given a link to the REDCap (Vanderbilt University) baseline survey, which included demographics and measures for caregiver burden, perceived stress, well-being, and perceived social support (Table 1). Surveys were collected at baseline (pretest) and at 2, 4, and 6 weeks (posttest) for the intervention and control groups. The control group was administered further surveys at weeks 8, 10, and 12. All participants received US $15 for every survey completed. Findings from the baseline and 6-week time points were collected to understand trends in study outcomes, while the 2- and 4-week surveys were collected to determine the feasibility of interim data collection. Participants in the intervention group received information about UpLode app access, which was the platform used to deliver the intervention. Participants in the control group were given an informational packet about stress management, mindfulness, and support resources at the start of pilot-testing, and then they received access to the app-based intervention after the 6-week data collection time point. All study information and procedures were available in English and Spanish in verbal and written forms, including written study materials. Surveys were translated into Spanish by a bilingual study team member and/or a certified Spanish translation service. Study meetings and email contact with Spanish-speaking participants also took place with a bilingual study team member.

**Table 1.** Data collection tools and time points for Latino millennial caregivers.

Tool	Time point
Demographics	Baseline for everyone
ZBI-12^a^	Intervention: baseline and 2, 4, and 6 wk; control: baseline and 2, 4, 6, 8, 10, and 12 wk
PSS^b^–Short Form	Intervention: baseline and 2, 4, and 6 wk; control: baseline and 2, 4, 6, 8, 10, and 12 wk
Neuro-QOL PAW^c^–Short Form	Intervention: baseline and 2, 4, and 6 wk; control: baseline and 2, 4, 6, 8, 10, and 12 wk
PROMIS^d^ ES^e^ and IS^f^ scales	Intervention: baseline and 2, 4, and 6 wk; control: baseline and 2, 4, 6, 8, 10, and 12 wk
Acceptability	Intervention: 6 wk; control: 12 wk

a ZBI-12: Zarit Burden Interview–Short Form.

b PSS: Perceived Stress Scale.

c Neuro-QOL PAW: Quality of Life in Neurological Disorders positive affect and well-being scale.

d PROMIS: Patient-Reported Outcomes Measurement Information System.

e ES: emotional support.

f IS: informational support.

### Intervention

This intervention was iteratively designed with feedback from millennial caregivers broadly and Latino millennial caregivers specifically and was guided by 2 frameworks: the stress process model and the dynamic fit model [21,33-35]. The stress process model explains how ongoing caregiving demands, especially when combined with limited resources, can lead to emotional distress and health outcomes such as anxiety and depression over time [35]. It highlights how resources and support can buffer the effects of stressors, including family caregiving. On the basis of caregiver feedback and this model, a resource list and support tools were included in the intervention design [33]. The dynamic fit model emphasizes that stress management and emotional regulation strategies are more effective when they match the demands of the stressful situation [21]. The intervention design includes mindfulness as an in-the-moment strategy and journaling as a sustained strategy to manage stress and emotional regulation (see Table 2 for more details). In alignment with the dynamic fit model, stress and caregiver burden were our primary study outcomes.

**Table 2.** Stress management and emotional regulation intervention components.

Intervention component	Description
Mindfulness activities	Step-by-step or audio instructions for: mountain meditation, loving-kindness meditation, STOP^a^, and mindful breathing meditation
Journaling activities	Journal prompts: What was one good event today? Realize if you played a role, you can most likely cause this to happen again. If you had no role, remember to express gratitude for this gift. What was your role in the event? What was one challenge you experienced today?
Emotion checklist	How did you feel (amazed, amused, excited, happy, peaceful, satisfied, anxious, ashamed, confused, sad, scared, upset)?
Stress rating scale	On a scale of 1 to 10, please rate your level of stress at this moment: 1=Low, 10=High
Caregiver support plan	The caregiver fills out the following plan: warning signs of stress or burden, activities I can do to help with stress, people I can call for help, ways that people can help me, my personal strengths that can help me, and numbers I can call in a crisis
Resources	Advance care planning Disease-focused information Caregivers of veterans Caregiver well-being Caregiving, culture, and faith Creating a medication list or medication education Community resource guide Employment and caregiving Family meetings Finances and caregiving Hospital discharge planning Insurance Long-distance and sandwich caregiving Physical care tips Preparing for a health care appointment Prescription assistance programs Safety Social support State-by-state resources
Scheduled reminder	Participants can opt for a reminder for intervention activities at a time that works best for them

a STOP: stop, take, observe, and proceed.

Our fully automated intervention was developed using iterative refinement based on feedback from a broad millennial family caregiver population followed by feedback from Latino millennial caregivers, and then it was tested for usability in both English and Spanish [33,34]. Through interviews with Latino millennial caregivers, the need for practical social support was highlighted, including education, more tailored support resources by geography and culture, and ways to connect with other family caregivers. Additional suggestions included having more mindfulness options and flexibility with journaling content, including an emotions checklist [34]. By incorporating mindfulness, journaling, and practical support strategies, this intervention sought to address the diverse needs of caregivers (Table 2).

Study participants received instructions to complete 1 mindfulness activity per day and respond to the 3 journaling prompts daily. The mindfulness instructions were a protocol deviation. We originally planned for participants to complete at least 6 minutes of mindfulness, but through feedback from Latino millennial caregivers, we adjusted this to mindfulness activities of varying lengths. Participants were also given information on communicating with the app developer if they were having functionality issues with the app.

Pilot-testing of the intervention took place concurrently in the non-Latino and Latino populations, and the results from the non-Latino study are reported elsewhere. The trial architecture and instrument battery were shared across both study populations [36]. Study findings from the non-Latino group revealed slightly higher ratings for mindfulness on its use for stress. There were lower study retention rates in the intervention group. Regarding preliminary study outcomes, the intervention group did not experience significant improvement in burden and stress but did experience statistically significant improvements in positive affect and well-being, informational support, and emotional support [36].

### Sample and Setting

Participants were self-identified family caregivers (including family members, friends, and neighbors) of individuals with chronic illnesses or disabilities who were recruited nationally in the United States from August 1 to October 31, 2023. Recruitment took place with support from Trialfacts, a research recruitment company. Trialfacts assisted the research team with the development of advertising materials consistent with the goals of the study and approved by the research team and the institutional review board prior to distribution. Advertising materials were then distributed on social media and other advertising sites, including Facebook, Instagram, Trialfacts email database, YouTube, Craigslist, TikTok, and Google. All potential participants completed prescreening surveys, and then eligible caregivers were contacted by research staff for a study intake interview. Eligible caregivers met the following inclusion criteria: being born between 1981 and 1996; provision of care for a family member, neighbor, or friend with a chronic illness or disability for >10 hours per week; identifying as Latino, Latina, Latine, or Latinx; English or Spanish speaking; and having technology access for study procedures. A 2-step screening process was used to protect against fraudulent data due to online recruitment strategies (see Multimedia Appendix 1 for the fraud protocol). During the study intake interview, participants were screened for study inclusion and provided with information about the study through the consent form. During recruitment, potential participants provided feedback about “Latino” being the preferred term.

Mindfulness interventions generally result in small to medium effect sizes. To move to a larger trial with 80% power and a small to medium effect size, 10 to 20 participants per research arm were needed for this pilot study. Fifteen participants per research arm were recruited.

### Instruments

We collected age in years, race and ethnicity, gender identity, relationship status, educational level, employment status, annual income, and caregiving characteristics (time spent caregiving, relationship to the care recipient, and the care recipient’s age).

#### Primary Study Outcomes

##### Caregiver Burden: Zarit Burden Interview–Short Form

This 12-item scale measures burden related to caregiving [37]. Items are scored on a 5-point Likert scale. Scores range from 0 to 48, with higher scores meaning greater caregiver burden. Reliability has consistently been reported to be above 0.70, with a clinical cutoff of 11 or greater for high caregiver burden [38].

##### Caregiver Perceived Stress: Perceived Stress Scale–Short Form

This 10-item, 5-point Likert scale (0=never; 4=very often) assesses thoughts and feelings associated with stress over a 1-month period. Scale summed scores range from 0 to 40, with scores of 0 to 13 considered low stress, 14 to 26 considered moderate stress, and 27 to 40 considered high stress. The Cronbach α was 0.91 [39,40].

### Secondary Study Outcomes

#### Caregiver Well-Being: Neurology Quality of Life Positive Affect and Well-Being Scale–Short Form

This 9-item, 5-point Likert scale (1=never; 5=always) addresses well-being, hope, purpose, meaning, and balance. Summed scale scores range from 9 to 45 and can be standardized to a T-score. For the purposes of this study, a raw score was used. Higher scores indicate greater well-being. The Cronbach α ranged from 0.94 to 0.95 [41].

#### Caregiver Perceived Social Support: Patient-Reported Outcome Measurement Information System Emotional Support and Informational Support Scales

These are 4-item, 5-point Likert scales (1=never; 5=always) with questions about having a support person for listening, confiding in, and feeling appreciated on bad days (emotional support) and having a support person who provides advice during a crisis, useful advice about important things in life, suggestions for dealing with problems, and information when needed (informational support). For both scales, scores range from 4 to 20 and can be standardized to a T-score. For the purposes of this study, a raw score was used. Higher scores indicate greater perceived emotional and informational support, with a Cronbach α above 0.88 for both scales [42,43].

#### Acceptability

The acceptability survey was constructed to address 7 facets of acceptability introduced by Sekhon et al [44] using Likert-scale questions (0=“not true for me”; 10=“very true for me”): affective attitude, intervention burden, ethicality, intervention coherence, opportunity costs, perceived effectiveness, and self-efficacy. The work by Sekhon et al [44] was selected due to the ability to assess acceptability at a more granular level, particularly with 2 main intervention components (mindfulness and journaling).

### Data Collection

Survey data were collected using REDCap surveys in English and Spanish at baseline and 2, 4, and 6 weeks (Table 1) [45,46]. With the 6-week survey, an acceptability survey was also included for the intervention group. The control group completed an acceptability survey 2 weeks after using the intervention for 4 weeks (week 12). There was one open-ended response question that allowed participants to share additional feedback. Feasibility data were collected throughout recruitment and data collection, including the number of participants screened (opting in and out), the number of participants recruited per week, and exit surveys for participants dropping out, capturing perceived barriers and reasons for leaving, adherence to the intervention (1 mindfulness practice per day and response to 3 journal prompts per day obtained through back-end app data), and overall study retention rates.

### Data Analysis

Baseline and 6-week intervention survey data were analyzed using descriptive statistics and frequencies. Due to the pilot nature of the study and small sample size, no inferential statistics to test the effect of the intervention were assessed. This was a protocol deviation, with the original plan being to use repeated-measure ANOVA. We made this change due to the attrition rate and having less than 15 participants per arm for the analysis. Acceptability survey responses were analyzed using descriptive statistics. Feasibility data were summarized using counts and percentages. Due to the small sample size, qualitative data are presented descriptively without additional analyses. With the exception of exit surveys for individuals dropping out of the study, only data from participants who completed the 6-week survey were included in the final analysis. All reported findings are from the Latino millennial caregiver group, and no figures, quotes, or statistical values were shared with the non-Latino millennial caregiver group.

### Ethical Considerations

This study was approved by the University of Texas at Austin Institutional Review Board (STUDY00002382), and participants provided written informed consent (Multimedia Appendix 2) prior to taking part. Data were deidentified to protect participant privacy and confidentiality.

## Results

### Demographics

A total of 30 Latino millennial caregivers participated in the study, but 9 did not complete the 6-week postintervention data surveys (Figure 1). The demographics reported here include the 21 participants who completed the 6-week data surveys, with 10 in the intervention group and 11 in the waitlist control group (Table 3). The mean age of participants was 35 (SD 4.79) years. Most participants were female (15/21, 71.4%), and nearly half (10/21, 47.6%) were Black or African American individuals, followed by White individuals (5/21, 23.8%) and participants who selected the self-identification option for race (3/21, 14.3%). Nearly half (10/21, 47.6%) of the participants were married or partnered, and over half (11/21, 52.4%) worked full time. Educational levels varied, with 38.1% (8/21) holding a bachelor’s degree, 19.0% (4/21) holding a master’s degree, and the remainder reporting some college or high school education. The average annual income across the sample was US $49,697 (SD US $26,249). Care recipients were primarily parents or in-laws (9/21, 42.9%) and had a mean age of 56 (SD 24.11) years. Caregivers reported providing an average of 48.43 (SD 62.08) hours of care per week.

**Figure 1.**
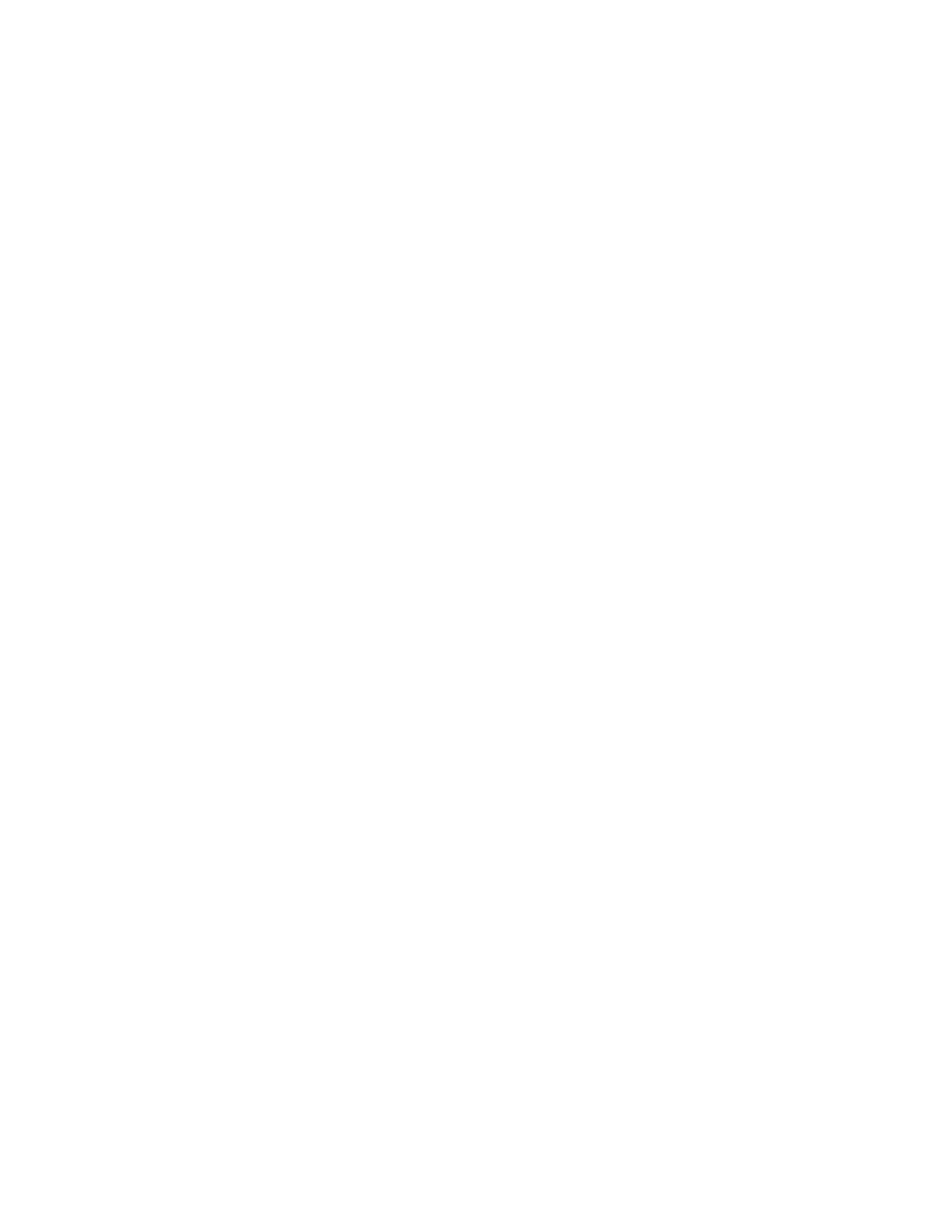
Stress management and emotional regulation study CONSORT (Consolidated Standards of Reporting Trials) diagram.

**Table 3.** Latino millennial caregiver participant demographics.

Demographic variable	Intervention (n=10)	Control (n=11)	Both (n=21)
Age (years), mean (SD)	35 (4.08)	36 (5.53)	35 (4.79)
Gender, n (%)			
Woman	7 (70)	8 (73)	15 (71)
Man	3 (30)	3 (27)	6 (29)
Race, n (%)			
American Indian/Alaska Native	0 (0)	1 (9)	1 (5)
Black/African American	7 (70)	3 (27)	10 (48)
White	1 (10)	4 (36)	5 (24)
Self-identify	1 (10)	2 (18)	3 (14)
Prefer not to answer	1 (10)	0 (0)	1 (5)
No answer	0 (0)	1 (9)	1 (5)
Marital status, n (%)			
Single	3 (30)	5 (46)	8 (38)
Partnered/Married	6 (60)	4 (36)	10 (48)
Divorced	0 (0)	1 (9)	1 (5)
Missing	1 (10)	1 (9)	2 (10)
Annual income (US $), mean (SD)	53,758 (27,276)	46,006 (26,019)	49,697 (26,249)
Educational attainment, n (%)			
High school/GED^a^	1 (10)	2 (18)	3 (14)
Some college	2 (20)	2 (18)	4 (19)
Associate’s degree	0 (0)	1 (9)	1 (5)
Bachelor’s degree	3 (30)	5 (46)	8 (38)
Master’s	3 (30)	1 (9)	4 (19)
Other	1 (10)	0 (0)	1 (5)
Employment, n (%)	2 (20%) 0 (0)		
Full-time	5 (50)	6 (55)	11 (52)
Part-time	2 (20)	3 (27)	5 (24)
Providing family care	2 (20)	0 (0)	2 (10)
Unemployed	0 (0)	1 (9)	1 (5)
Looking	1 (10)	0 (0)	1 (5)
Retired	0 (0)	1 (9)	1 (5)
Care recipient relationship, n (%)			
Child	1 (10)	2 (18)	3 (14)
Parent/In-law	5 (50)	4 (36)	9 (43)
Grandparent	0 (0)	2 (18)	2 (10)
Sibling	0 (0)	1 (9)	1 (5)
Other relative	2 (20)	2 (18)	4 (19)
Missing	2 (20)	0 (0)	2 (10)
Care recipient age (years), mean (SD)	56 (20.96)	56 (27.69)	56 (24.11)
Time spent caregiving (hours/week), mean (SD)	38 (56.94)	58 (67.70)	48.43 (62.08)

a GED: general educational development.

### Feasibility

Sixty-five potential participants completed the prescreening questionnaire, with 5 excluded due to not meeting the study criteria for age or number of caregiving hours. Of the 60 potential participants recruited for the study, 21 did not show up for the study intake interview, 4 failed the study intake interview (did not meet the study criteria), 1 declined to participate, and 1 met the criteria for fraud, and recruitment targets were met before the last 3 could complete intake interviews. We recruited the total sample of 30 participants in 12 weeks, with an average rate of 2.5 (SD 3.12) individuals per week. For data collection in weeks 2 and 4, a total of 24 and 21 participants completed the surveys, respectively. Nine participants did not complete the 6-week survey, although no participants explicitly dropped out. Therefore, the overall retention rate was 70% (21/30; 10/15, 66% in the treatment group and 11/15, 73.3% in the control group).

With the intervention itself, all 10 participants in the intervention group completed a mindfulness and journaling activity at least once. No participants met the threshold for completing all 84 daily journal entries (calculated as 3 journal prompts per day for 28 days). Six participants met the threshold of daily journal completion (range 32-65), with the remaining 4 ranging from 1-26 completed journal entries. Overall, participants completed an average of 33.65 (SD 22.97, range 1-65) journal entries, reflecting a 39.6% adherence rate based on the expected 84 entries. In contrast, only 3 (30%) individuals met the threshold for daily mindfulness completion (range 89-166), with the remaining participants (n=7, 70%) completing daily mindfulness between 0-14 days. Due to the wide range of mindfulness activity completion, participants completed an average of 37.9 (SD 57.96, range 0-166) mindfulness sessions overall. This suggests that some participants engaged in multiple mindfulness activities on certain days.

### Acceptability

Acceptability scores ranged from moderate to high, with participants generally expressing greater approval for the journaling activities compared to the mindfulness components. On a scale from 1 to 10, journaling was rated more favorably across multiple items: participants agreed more strongly that journaling helped manage stress (mean 7.00, SD 2.68) and was appropriate for their stress management needs (mean 7.46, SD 1.85) and felt that they could use it to help with stress (mean 7.92, SD 2.34). In contrast, mindfulness activities received lower ratings on helpfulness (mean 5.54, SD 3.97) and perceived appropriateness (mean 6.15, SD 3.08), although participants generally agreed that the activities made sense (mean 7.38, SD 2.36) and could potentially be used to manage stress (mean 6.69, SD 2.84; Multimedia Appendix 3).

### Preliminary Outcomes

For the control group, median burden levels were 18 before and after the intervention (IQR 13-23 and 10-23, respectively). Median perceived stress also remained the same before (median 15, IQR 11-20) and after the intervention (median 15, IQR 6-20). The median positive affect and well-being score was 37 (IQR 31-42) before the intervention and 41 (IQR 28-45) after the intervention. Median informational support scores increased from 14 (IQR 12-17) to 16 (IQR 13-18) from before to after the intervention. Median emotional support levels increased from 15 (IQR 12-20) before the intervention to 16 (IQR 13-19) after the intervention (Multimedia Appendix 4).

For the intervention group, the median burden level decreased from 20 (IQR 15-27.25) to 15 (IQR 10.50-28.75) from before to after the intervention. Median perceived stress increased from 19.50 (IQR 12-26.75) to 21 (IQR 8.75-22.75) from before to after the intervention. Positive affect and well-being and informational support median scores also increased from 33 (IQR 21.75-38) before the intervention to 34.50 (IQR 23-43) after the intervention and from 12 (IQR 10-16.50) before the intervention to 14 (IQR 11.75-17) after the intervention, respectively. Finally, emotional support marginally decreased from 14.5 (IQR 11.5-17.75) before the intervention to 14 (IQR 12-17.25) after the intervention.

## Discussion

### Principal Findings

This pilot study evaluated the feasibility, acceptability, and preliminary outcomes of an mHealth intervention designed to support stress management among Latino millennial caregivers. The study demonstrated successful recruitment completed within 12 weeks and a 70% (21/30) overall retention rate. Study adherence, particularly with mindfulness activities, was low. Acceptability findings mirrored adherence, with participants generally favoring journaling activities over mindfulness components, citing journaling as more helpful and easier to integrate into daily routines.

The findings of this study provide preliminary insights into the differential acceptability of journaling- and mindfulness-based interventions among Latino millennial caregivers. Adherence and acceptability findings, particularly related to mindfulness activities, demonstrate that more work needs to be done with the mindfulness component of this intervention. Although we did build the intervention with feedback from Latino millennial caregivers, it is evident that additional feedback and intervention iteration are needed. Our operationalization of mindfulness may have lacked the depth of cultural adaptation observed in other studies, such as that by Juarez-Reyes et al [47], where a Spanish-delivered mindfulness-based intervention for Latino patients with cancer achieved high feasibility and acceptability through metaphors, trauma-informed approaches, and spiritual acknowledgment. This suggests that while mindfulness has evidence of effectiveness in prior studies, its uptake among Latino caregivers may hinge on tailored adaptations that reflect lived experiences and values. On the basis of our acceptability findings, caregivers may also need options that are brief, lasting from 1 to 2 minutes rather than 3 to 6 minutes. By expanding the types and lengths of activities, there may be better future uptake.

The relatively higher ratings and qualitative endorsements of journaling by participants in our study are consistent with the literature on writing therapy. Ruini and Mortara [24] describe journaling as an effective, stand-alone intervention that enhances self-awareness, insight, and emotional regulation. Given the multiple roles and responsibilities reported by caregivers, journaling may offer a better fit due to its flexibility, brevity, and individual tailoring, qualities also emphasized in prior studies as essential for mHealth success [32]. Additionally, voice-to-text as well as written text were available with this intervention, offering caregivers multiple methods for journal entry. Due to our adherence findings, future intervention iterations may benefit from offering one daily journal question rather than three.

The feasibility and preference differences for journaling and mindfulness may also reflect cultural and contextual constraints. Prior studies have reported time constraints, mental exhaustion, and the need for culturally relevant delivery as common barriers among Latino caregivers [48]. Participants in our study echoed these barriers, particularly in relation to mindfulness activities. This is supported by Manzo et al [49], who found that Latino caregivers implementing mindfulness techniques such as mindful breathing showed gradual adoption over 5 weeks but still faced challenges in integrating practices consistently due to caregiving demands.

We did not conduct statistical testing to assess intervention efficacy. The preliminary outcome trends highlight important areas for consideration that may enhance understanding of feasibility and acceptability findings. The primary study outcomes highlight areas for intervention iteration. Median burden scores decreased in the intervention group and remained the same in the control group. In the intervention group, perceived stress increased from before to after the intervention, while stress remained the same in the control group. This may align with our findings that the intervention takes too much time, thus increasing stress. As noted earlier, shorter mindfulness options and fewer journaling questions may offer a less stressful and time-consuming intervention. These findings might also be a reflection of how the different intervention activities were received, with participants favoring journaling over mindfulness. Mindfulness as an in-the-moment response to stress may map onto perceived stress more readily than caregiver burden, while journaling may map onto caregiver burden more readily, with it being a way to process positive experiences and challenges. In future studies, we will test the intervention components independently or use a multiphase optimization strategy to determine the effects of intervention components on study outcomes and whether the intervention components are more effective delivered alone or together [50]. Bringing the findings back to the community will also help us determine next steps that remain in alignment with Latino millennial caregivers’ needs.

Secondary outcome patterns also highlight important considerations for intervention iteration and testing. Both groups showed an increase in informational support. The intervention group received resources within the app, and the control group received resources in written form. For future intervention iteration and testing, a different attention control option may be needed. It may be that receiving support is important regardless of the form of delivery. Positive affect and well-being also improved in both groups, which may also relate to participants in both groups receiving resources, although in different forms. Emotional support increased in the control group and decreased, although marginally, in the intervention group. This pattern may reflect the combination of both groups receiving resource support but the intervention group also experiencing increased stress with the intervention activities. For future intervention iteration and testing, both engaging with Latino millennial caregivers regarding our study findings and using the multiphase optimization strategy will provide a clearer picture of which intervention components are best suited to support Latino millennial family caregivers’ stress, burden, well-being, and support outcomes [50].

### Study Limitations

Our study has important limitations that affect the generalizability of our findings. The first limitation is our small sample size and lack of intervention efficacy testing; therefore, inferences about intervention efficacy cannot be made. Alongside the small sample, we had a high study attrition rate, which highlights potential flaws or barriers with study implementation. The second limitation is the accessibility and generalizability of a digital intervention with existing inequities in mobile phone and broadband internet access. This is a notable concern, yet demographic trends in the United States support high use of smartphones in Latino adults (93% of Latino adults vs 91% of non-Hispanic White adults), with 75% having home broadband and 20% being dependent on a smartphone for going online. An estimated 97% of millennial adults own a smartphone, which aligns with the age demographic for this study [51,52]. This leaves a small proportion of Latino millennial caregivers who would not be able to access this intervention; such a gap can be addressed in larger studies with the provision of study phones for intervention access. The third limitation is the demographic representativeness of the study population. Our sample had a higher proportion of women, Black individuals, and well-educated participants. In other mHealth studies, there has been higher adoption by participants who were women. Many studies have an overrepresentation of White participants, while ours had a disproportionately higher number of Latino Black participants. Finally, educational level can be a major barrier to health care access, health education, and intervention adoption. Our recruitment approach may have limited the educational diversity of participants, which is an important issue to address in future work.

### Conclusions

Our study findings suggest important gaps related to mindfulness acceptability and participant retention rates that will require feedback from Latino millennial caregivers and further intervention iteration.

## Supplementary material

10.2196/85396 Multimedia Appendix 1 Fraud protocol for participant recruitment and enrollment.

10.2196/85396 Multimedia Appendix 2 Consent form.

10.2196/85396 Multimedia Appendix 3 Acceptability results.

10.2196/85396 Multimedia Appendix 4 Median scores and IQRs for the intervention and control groups.

10.2196/85396 Checklist 1 CONSORT-EHEALTH checklist (V 1.6.1).

## Abbreviations

CONSORT-EHEALTH: Consolidated Standards of Reporting Trials of Electronic and Mobile Health Applications and Online Telehealth
mHealth: mobile health
