# Supplementary material for: An mHealth Stress Management Intervention for Latino Millennial Caregivers: Pilot Feasibility Randomized Controlled Trial
Source: JMIR Form Res. 2026 Oct 27;10:e85396. doi: 10.2196/85396 (PMC13717342; doi:10.2196/85396)
Supplement: Supplementary file 3 [file formative-v10-e85396-s002.docx]

**Consent to Participate in Research**

**Basic Study Information**

Title of the Project: Cultural and Language Adaptation of a Stress, Emotional Regulation, and Support Intervention in Latinx Millennial Caregivers.

Principal Investigator: Megan Thomas Hebdon, PhD, DNP, RN, NP-c; University of Texas at Austin School of Nursing

**Invitation to be Part of a Research Study**

You are invited to be part of our research study, *Cultural and Language Adaptation of a Stress, Emotional Regulation, and Support Intervention in Latinx Millennial Caregivers.* This consent form will help you choose whether or not to participate in the study. Feel free to ask if anything is not clear.

**What is the study about and why are we doing it?**

The purpose of the study is adapt an existing phone app and intervention for Millennial caregivers. We want to make changes so that it will meet the language, stress, support, and emotional needs of Latinx Millennial caregivers. Then, we want to test it in Latinx and non-Latinx Millennial caregivers.

**What will happen if you take part in this study?**

If you agree to take part in this study, you will be asked to do one of three, or possibly all three of the following things:

1. A focus group interview through Zoom or in person with other Latinx Millennial caregivers that will take no more than 90 minutes. The audio from the interview will be recorded and transcribed. Any identifying information about you will be removed.
2. Review an mHealth app for content and clarity through Zoom. Your feedback will be written down, but none of your personal information will be collected.
3. Participate in a 6- or 12-week intervention. You will be randomized to one of two groups to receive a stress, emotional regulation, and support intervention using a mobile health app. This should take no more than 30 minutes each day. You will be asked to complete a daily question (about 1 minute) and surveys before the intervention starts and weeks 2, 4, and 6 (8, 10, and 12 depending on the study arm) through a mobile app. These surveys should take no more than 20 minutes.

**How long will you be in this study and how many people will be in the study?**

Depending on which part(s) of the study you do, the number of people and the length of time will be different.

1. If you participate in the focus group interview, this will take no more than 90 minutes, including a short demographic survey at the beginning. Up to 30 people will participate in the focus group interviews.
2. If you review the mHealth app, this will take no more than 60 minutes. If you have not already completed a demographic survey, you will be asked to do this. Three people will participate in this step.
3. If you participate in the intervention, your participation may take 6 or 12 weeks depending on the group you are randomized to. Up to 30 people will participate in this step. You will be asked to respond to a question in the UpLode app every day and participate in the intervention every day. The intervention should take no more than 20 minutes. You will be asked to complete surveys in the UpLode app before the intervention starts and at weeks 2, 4, and 6 (8, 10, and 12 depending on the study arm). These surveys should take no more than 20 minutes.

**What risks and discomforts might you experience from being in this study?**

If you participate in this study, you will experience no more risk than you would in daily life. You may experience distress when discussing your experiences as a family caregiver. If you do, you can stop answering questions or discontinue the study at any time.

**How could you benefit from this study?**

Although you will not directly benefit from being in this study, others might benefit because what you share about your experiences as a family caregiver will increase our understanding about Millennial Latinx caregivers. In addition, if you participate in the intervention, you will inform the process and delivery of the intervention for caregivers in the future.

**What will happen to the data we collect from you?**

As part of this study we will collect data from an online demographic survey. Depending on your participation in other steps, we will record the audio from a focus group interview, take notes during feedback about an app and intervention, collect a daily question and/or collect surveys every about stress, well-being, and social support. We will store this data securely, analyze it, and report any findings in a group format.

**How will we protect your information?**

To protect your privacy, we will collect information that could directly identify you for study communication purposes only including name, phone, and/or email. We will protect your information by only collecting data that is necessary for our research, including emailing you a gift card or emailing/calling you to set up an interview. This information will be stored separately from other study data including interviews or answers to questionnaires.

We will ask you if you are willing to be re-contacted for future studies by our research team. If you say yes, we will store your contact information (name, phone and/or email) for future studies. If you say no, your contact information will be destroyed after study completion. Your contact information will be stored separately from your other data. All of your data will be stored in a password protected file.

We plan to publish the results of this study. To protect your privacy, we will not include any information that could directly identify you. We will share your data or samples with other researchers for future research studies that may be similar to this study or may be very different. The data or samples shared with other researchers will not include information that can directly identify you.

If the research team sees or hears anything that indicates a child or elder is being abused, we are required by law to report such abuse.

**What will happen to the information we collect about you after the study is over?**

We will keep your research data to use for future analyses. Your contact information will be kept securely and stored separately from the research data collected as part of the project. We will destroy your contact information if you opt out of being re-contacted for future studies. Your contact information will be stored securely for future studies if you opt in to being re-contacted.

**How will we compensate you for being part of the study?**

You will be compensated differently depending on which parts of the study you do.

1. If you do the focus group interview, you will receive a $70 Tango card after the interview. You must attend at least half of the focus group to receive compensation. If you do not, you will offered the option to complete the questions with a subsequent focus group interview or one-on-one interview. To receive a $70 Tango card, you must attend at least half of the one-on-one interview.
2. If you provide feedback about the app and intervention, you will receive a $30 Tango card after providing feedback.
3. If you participate in the intervention, you will receive between $5-$15 in compensation each week, depending on survey length for that week. If you complete at least one daily question per week, you will receive $5. You will receive $15 for the baseline survey, and surveys at weeks 2, 4, and 6 (8, 10, and 12). Depending on the group you are assigned to, you will receive between $70-$125 in total compensation through Tango cards.

**Your Participation in this Study is Voluntary**

It is totally up to you to decide to be in this research study. Participating in this study is voluntary. Your decision to participate will not affect your relationship with The University of Texas at Austin. You will not lose any benefits or rights you already had if you decide not to participate. Even if you decide to be part of the study now, you may change your mind and stop at any time. You do not have to answer any questions you do not want to answer.

**Contact Information for the Study Team**

If you have any questions about this research, or feel you may have been harmed due to participation, you may contact:

Dr. Megan C. Thomas Hebdon

Phone: 512-475-9718

Email: megan.hebdon@nursing.utexas.edu

**Contact Information for Questions about Your Rights as a Research Participant**

If you have questions about your rights as a research participant, or wish to obtain information, ask questions, or discuss any concerns about this study with someone other than the researcher(s), please contact the following:

The University of Texas at Austin Institutional Review Board

Phone: 512-232-1543

Email: irb@austin.utexas.edu

Please reference the protocol number found at the top of this document.

**Your Consent**

By signing this document, you are agreeing to be in this study. We will give you a copy of this document for your records. We will keep a copy with the study records. If you have any questions about the study after you sign this document, you can contact the study team using the information provided above.

*I would like to opt out of my contact information being saved for future studies.* Yes	No

*I understand what the study is about and my questions so far have been answered. I agree to take part in this study.*

______________________________________________________________

Printed Subject Name

______________________________________________________________

Signature	Date
