# Supplementary material for: An mHealth Stress Management Intervention for Latino Millennial Caregivers: Pilot Feasibility Randomized Controlled Trial
Source: JMIR Form Res. 2026 Oct 27;10:e85396. doi: 10.2196/85396 (PMC13717342; doi:10.2196/85396)
Supplement: Supplementary file 4 [file formative-v10-e85396-s003.docx]

Appendix 3: Study Acceptability Findings

| **Question** | **M** | **SD** | **Range** |
| --- | --- | --- | --- |
| I liked the mindfulness activities. | 6.69 | 2.84 | 0-10 |
| I liked the journaling activity. | 7.08 | 2.60 | 0-10 |
| The mindfulness activities took too much time. | 4.08 | 3.15 | 1-10 |
| The journaling activity took too much time. | 4.31 | 3.01 | 1-10 |
| The mindfulness activities were appropriate for my stress management needs. | 6.15 | 3.08 | 1-9 |
| The journaling activity was appropriate for my stress management needs. | 7.46 | 1.85 | 3-10 |
| The mindfulness activities made sense to me. | 7.38 | 2.36 | 3-10 |
| The journaling activity made sense to me. | 7.77 | 2.01 | 5-10 |
| The mindfulness activities helped my stress. | 5.54 | 3.97 | 0-10 |
| The journaling activity helped my stress. | 7 | 2.68 | 0-10 |
| I feel like I can use the mindfulness activities to help my stress. | 6.69 | 2.84 | 1-10 |
| I feel like I can use the journaling activity to help my stress. | 7.92 | 2.34 | 2-10 |
| Please share any other feedback about the intervention. | Positive:  The intervention was so nice. Providing the audio part of the mindfulness activities helped do the activities with ease. They were well timed and well organized.  Journaling was good to identify my stressors and realize then on bad days, there is something I can be grateful about. It'd be nice to get the log of those, so I can better see what needs to change.  Negative:  I might be in the "control group" for those who didn't do mindfulness, because it was nearly impossible to find 5-6 minutes to just sit in silence. I could have made the time, but with so much that has to be done, and the mental exhaustion, doing the activity never seemed appealing enough.  I don't ever have the time or energy. | | |
