# Supplementary material for: An mHealth Stress Management Intervention for Latino Millennial Caregivers: Pilot Feasibility Randomized Controlled Trial
Source: JMIR Form Res. 2026 Oct 27;10:e85396. doi: 10.2196/85396 (PMC13717342; doi:10.2196/85396)
Supplement: Supplementary file 5 [file formative-v10-e85396-s004.docx]

Appendix 4: Stress Management and Emotional Regulation Study Preliminary Outcomes

| Variable/  Time Point | Intervention (n=10)  Median (IQR) | Control (n=11)  Median (IQR) |
| --- | --- | --- |
| Zarit Burden Interview pre-test | 20 (15-27.25) | 18 (13-23) |
| Zarit Burden Interview post-test | 15 (10.50-28.75) | 18 (10-23) |
| Perceived Stress Scale pre-test | 19.50 (12-26.75) | 15 (11-20) |
| Perceived Stress Scale post-test | 21 (8.75-22.75) | 15 (6-20) |
| Positive Affect and Well-Being pre-test | 33 (21.75-38) | 37 (31-42) |
| Positive Affect and Well-Being post-test | 34.50 (23-43) | 41 (28-45) |
| Informational Support pre-test | 12 (10-16.50) | 14 (12-17) |
| Informational Support post-test | 14 (11.75-17) | 16 (13-18) |
| Emotional Support pre-test | 14.5 (11.5-17.75) | 15 (12-20) |
| Emotional Support post-test | 14 (12-17.25) | 16 (13-19) |

IQR=Interquartile Range
